# Boxing with and without Kicking Techniques for People with Parkinson’s Disease: An Explorative Pilot Randomized Controlled Trial

**DOI:** 10.3233/JPD-223447

**Published:** 2022-12-16

**Authors:** Josefa Domingos, Ana Ligia Silva de Lima, Tessa Steenbakkers-van der Pol, Catarina Godinho, Bastiaan R. Bloem, Nienke M. de Vries

**Affiliations:** a Radboud University Medical Center, Donders Institute for Brain, Cognition and Behaviour, Department of Neurology, Center of Expertise for Parkinson and Movement Disorders, Nijmegen, The Netherlands; b Grupo de Patologia Médica, Nutrição e Exercício Clínico (PaMNEC) do Centro de Investigação Interdisciplinar Egas Moniz (CiiEM), Monte de Caparica, Portugal

**Keywords:** Parkinson’s disease, boxing, exercise, balance, kicking, physiotherapy

## Abstract

**Background:**

People with Parkinson’s disease (PD) benefit from boxing exercise. Adding kicking variations to the boxing may provide additional benefit to improve balance. However, the benefits and adherence to such trainings is unknown.

**Objective:**

To explore the feasibility, safety, and benefits on balance of boxing training combined with kicking techniques in comparison to boxing without kicking in PD.

**Methods:**

Participants were randomized to group-based boxing training with kicking techniques (BK) or to group-based boxing alone training (BO). Both groups trained for one hour, once a week, for a period of 10 weeks. Participants were assessed at baseline and ten weeks post-intervention for difference in balance, fear of falling, balance confidence, walking ability, and quality of life.

**Results:**

Twenty-nine people with PD (median age 64 years; median disease duration 5 years) participated. Both interventions were feasible and acceptable for all participants. No adverse events occurred. Most participants (BK 80%; BO 75%) were satisfied with the training. We found no significant between group difference on either the primary (Mini-BEST) or secondary outcomes. The within group comparison showed that balance improved in both groups after the intervention (BK 22.60 (2.7) to 25.33 (2.64) *p* = 0.02; BO 23.09 (3.44) to 25.80 (2.39); *p* = 0.01 on the Mini BEST test).

**Conclusion:**

Both types of boxing seem to be feasible and safe. Adding kicking techniques to boxing does not improve balance significantly more than boxing alone. Incorporation of kicking may be a valuable addition to the exercise therapy repertoire.

## Plain Language Summary

Boxing appears to be an effective treatment for persons with Parkinson’s disease. Thus far, studies mostly looked at punching movements of the arms, but this new research examined whether adding additional kicking movements with the legs would add further benefits. The researchers were also interested whether the addition of kicking movements would make the exercise program more palatable for the participants, leading to a better compliance. Twentynine persons with Parkinson’s disease participated in boxing classes for a total duration of 10 weeks, and they were divided into two groups: punching with the arms only; or punching combined with kicking of the legs. Both types of boxing were feasible and acceptable for all participants. No adverse events occurred. Balance improved in both groups after the boxing classes, but the benefits were equal for both types of boxing. It is therefore not necessary to include kicking movements to gain greater clinical benefits from boxing, but incorporating such kicking movements remains potentially valuable as a way to make the exercise repertoire more versatile for participants.

## INTRODUCTION

People with Parkinson’s disease (PD) often present with gait and balance impairments [1, 2] and have a tendency towards an inactive lifestyle [3–6]. As such, people with PD benefit from several nonpharmacological approaches to prevent balance-related problems and to promote a physically active lifestyle.

Evidence regarding the effectiveness of different types of exercise in PD is accumulating [7–11]. In addition to the more traditional types of exercise, such as balance, resistance and endurance training [9, 11], alternative training methods are gaining popularity [11–14]. Boxing is one such popular and novel alternative training methods. Just as in other medical conditions (i.e., obesity [15] or stroke [16]), preliminary studies have demonstrated the some positive effects of boxing on balance, mobility, and quality of life in people with PD [17–20]. Another study showed that self-perceived QOL improvements were maintained throughout 20 weeks of non-contact group-based boxing training, despite lack of improvements in disease severity [21]. Even though the safety and long-term adherence of community-based group boxing for PD have been described previously [22], concerns rise regarding its implementation and the significant mismatch between the positive grandiloquence on online information websites about boxing for PD versus the concretely available research evidence. Importantly, there is still limited evidence on the efficacy, safety, disease-specific modifications, limitations, and health professional training needed [23].

Recent research also shows that offering group-based PD-specific boxing programs that are adaptable, varied, open to input, and that encouraging social support and networking may be beneficial to increasing motivation for exercise and physical activity in PD [24]. Boxing classes commonly consist of a warm-up, boxing specific exercise (boxing drills, shadow boxing, speedbag drills, jumping jacks, and strength exercises) and a cool down with stretching at the end [17–21]. Balance-demanding exercises (e.g., weight shifting, dynamic changes in balance, and postural adjustments) may induce synergistic effects for balance gains [9, 25]. As such, we believe that adding kicking techniques to boxing exercises may lead to a greater improvement in balance outcomes than boxing that only uses punches. However, in clinical practice, physiotherapists and trainers may be reluctant to add kicking techniques, which inevitably necessitates the participant to stand on one leg, challenging their balance, because of safety issues and potential fall risks. In PD, there is progressive reduction in the ability to control anticipatory postural adjustments prior to lifting one leg [26, 27] and an increased risk of falls associated to less than 10s one leg stance [28, 29].The possible benefits of kicking, its safety, feasibility and its acceptability have never been studied. The primary aim of the current exploratory study was to explore the feasibility, safety, and potential additional benefit of boxing training combined with kicking techniques on balance in comparison to boxing without kicking for people with PD.

## METHODS

We performed a single-blinded exploratory pilot randomized controlled trial. We included men and women with (self-reported) PD with sufficient knowledge of the Dutch language to complete the clinical tests and follow instructions during the training sessions. We excluded individuals who were unable to walk without using an assistance device or had significant cognitive impairment (i.e., Mini-Mental State Examination of < 24) [30].

This trial was conducted in compliance with the Ethical Principles for Medical Research Involving Human Subjects, as defined in the Declaration of Helsinki. The study protocol was evaluated by the local Commission Human research (CMO) Arnhem/Nijmegen (dossier number: 2019-5658). Before inclusion, all participants signed an informed consent form.

### Procedures

Participants were contacted through posters and flyers in a local hospital and a local physiotherapy practice. Twenty-nine participants volunteered and were, after screening for eligibility and baseline testing, randomly assigned to group-based boxing training with addition of kicking techniques (BK) or group-based boxing alone training (BO). An independent researcher performed the randomization in a 1 : 1 ratio using the data management system Castor (Electronic Data Capture, The Netherlands). Both groups trained once a week, one-hour per session, for a period of 10 weeks. No blinding was applied for the trainers and participants. The measurements were performed by a blinded researcher of the Radboudumc at baseline (T0), and after ten weeks (T1).

### Intervention

Training sessions for both groups were led by one physiotherapist specialized in PD (part of the nationwide Dutch ParkinsonNet) [31] and one boxing trainer to guarantee safety and assure appropriate training. Participants trained together in their respective groups with the same instructors. For the first group (BO), each session consisted of training punching movements and progressively adding in dual task challenges (i.e., going through a variety of punching combinations). The intensity and variety of exercises were increased according to the patient’s capacity every week. The second group (BK) received 10 sessions of group-based boxing training comparable to the previous group, but with added kicking techniques, weight shifting exercises and multidirectional stepping. A complete overview of the training sessions can be found in Supplementary Table 1.

**Table 1:** Demographic and clinical characteristics of both Boxing with Kicking and Boxing Alone group

	Boxing with Kicking *N* = 15	Boxing Alone *N* = 14	*p*
Age*	63.69 (SD 6.63)	64.36 (SD 11.14)	0.98
Sex (men)^†^	73% (*n* = 8)	60% (*n* = 6)	0.17
Amount of education (y)*	15.18 (SD 6.5)	15.30 (SD 5.14)	0.94
Time since first symptoms (y)*	9.09 (SD 5.73)	6.10 (SD 4.72)	0.14
Disease onset*	5.82 (SD 4.16)	4.26 (SD 4.58)	0.13
Dopamine replacement medication (yes)^†^	13	10	1.00
Affected side^†^	0.85
–Left	18% (*n* = 2)	30% (*n* = 3)
–Right	64% (*n* = 7)	50% (*n* = 5)
–Symmetrical	18% (*n* = 2)	20% (*n* = 2)

Missing values varies per variable and per group. *Mean (Standard deviation). ^†^Percentage. All percentages reported are over the total of responders.

### Outcome measurements

We collected demographic information on age, sex, and years of education. Additionally, we registered years since diagnosis, dopaminergic medication use, and most affected side. We assessed feasibility (adherence to training, satisfaction, perceived benefits, recommend to others) and safety (adverse events) using a self-administered questionnaire assessing participants experiences with the training. The complete questionnaire used can be found in the Supplementary Material.

We also assessed effectiveness of the trainings. The primary outcome measure was balance as measured by the Mini-Balance Evaluation Systems Test (Mini-BESTest), which assesses four balance subdomains (anticipatory postural adjustments, postural responses, sensory orientation, and stability in gait) [32]. The maximum score is 28 points, and each item is scored from 0 (unable or requiring help) to 2 (normal). As secondary outcomes, we measured fear of falling with the Falls Efficacy Scale International FES-I [33] and the Activities Balance Confidence Scale (ABC-scale) [34]. The ABC is a 16-item questionnaire designed to measure balance confidence in various everyday activities. For each item the score ranges from 0 to 10 points (total score will range from 0–160 points). The FES-I is a self-efficacy and balance confidence questionnaire, with 16 items. The score ranges from minimum 16 (no concern about falling) to maximum 64 (severe concern about falling). These were collected by sending out e-mails by the certified data management system castor EDC (electronically) when possible and on paper/pencil when participants preferred.

For walking ability, we used the 6-minute walk distance (6MWD) [35] and the Timed-Up-Go [36]. We also assessed health status and quality of life using the Parkinson’s Disease Questionnaire (PDQ-39). We selected all measurement instruments according to recommendations in the European Physiotherapy Guideline for Parkinson’s disease [9].

### Data analysis

We used the intention-to-treat principle in all analyses. The analysis was performed using IBM SPSS statistics version 26 (IBM, New York, United States). Shapiro-Wilk test was used to determine whether the data were normally distributed. Since the data were not normally distributed (*p* < 0.05), we used non-parametric tests for further analysis. Mann Whitney U tests was used to test the difference between the two groups concerning balance, walking ability, and fear of falling, follow-up (T1). Secondarily, we used the Wilcoxon Signed-rank test to evaluate differences within groups. The level of significance was set to p < 0.05. To measure the effect-size, we calculated Cohen’s d. The effect size can be either small (0.20), medium (0.50), or large (0.80) [37]. We used descriptive statistics and analysis of content to evaluate the narrative of participants collected via the qualitative questionnaires.

## RESULTS

### Participants

Twenty-nine people with PD (median age 64 (8.8) years; median disease duration 5 (4.3) years, time from first symptoms 7.5 (5.2) years) participated in this pilot explorative randomized controlled trial.

**Fig. 1:**
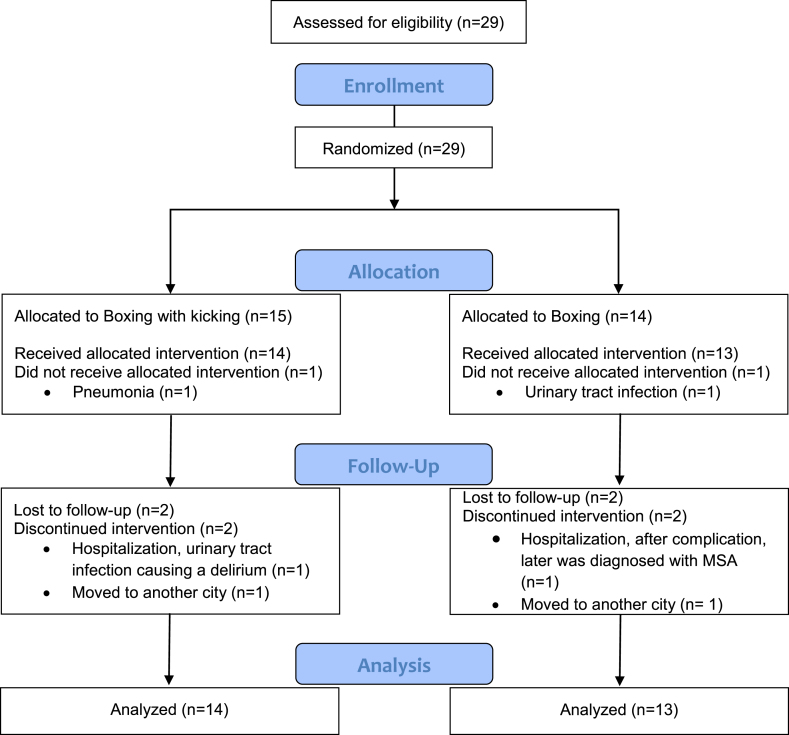
Flowchart of the study participants.

Both groups were comparable at baseline (*p* > 0.05; Table 1). Fourteen individuals participated in the boxing with kicking (BK), and thirteen in the boxing-alone (BO). From those, twelve participants in the BK and eleven in the BO group had a complete dataset (Fig. 1).

### Safety and feasibility

The trainings were completed by 85% of the participants. During the trial, only four dropouts occurred, all for reasons unrelated to the program. There were no fall incidents or other adverse effects events reported during training in both groups.

### Participants acceptability and perceived benefits

A total of 18 participants (*n* = 10 BK group and *n* = 8 BO group) replied to the qualitative questionnaire (Table 3). The majority (*n* = 8, 80%) of participants in the BK group reported satisfaction with the training and that they had improved their balance after training. These subjective improvements in both balance and exercise capacity were reported as a motivating factor to keep practicing boxing by 60% (*n* = 6) of the participants. When asked what the benefits of kicking techniques would be, most participants (60%, *n* = 6) replied that they expected that BK would improve their balance more than conventional boxing. All participants in the BK group (100%, *n* = 10) would recommend BK training to other people with PD.

Participants in the BO group reported similar improvements. Satisfaction and balance improvements were reported by 75% (*n* = 6) of participants in the BO group. Motivations to attend BO training were either improvements in balance and exercise capacity (50%, *n* = 4) or social contact (50%, *n* = 4). The two most mentioned benefits of BO training were: improvement in exercise capacity (37%, *n* = 3) and improvement in mobility (25%, *n* = 2). All responders (*n* = 8) in the BO group would recommend BO to other people with PD.

**Table 3:** Participants experience and perceived benefit of both Boxing with kicking and Boxing group

	Boxing with Kicking *N* = 10	Boxing Alone *N* = 8
Satisfaction	80%	75%
Perceived improvements in balance	8	6
Areas of motivation to keep boxing	Improvements in balance and exercise capacity	Improvements in balance and exercise capacity or social contact
Would recommend to others	All	All

### Balance

We did not find any additional benefits of kicking techniques (betewen group differences, BK 25.33 (2.64) and BO 25.80 (2.39) *p* = 0.53).

The within-group comparison showed that balance improved in both groups after the intervention (BK 22.60 (2.7) to 25.33 (2.64) *p* = 0.02; BO 23.09 (3.44) to 25.80 (2.39); *p* = 0.01 on the Mini BEST test) (Table 2). The effect size of both types of boxing modalities was large, 1.02 for BK and 0.91 for BO for the Mini-BESTest. Results showed that four people in the BK group and 4 in the BO group improved at least four points in the Mini-Best score, which has been reported as the Minimally Clinical Important Difference) [38].

### Fear of falling

Fear of falling, as measured by the FES-I, did not show a significant between group difference after the intervention (BK 27.36 (6.56) and BO 24.00 (3.97) *p* = 0.34).

Within group comparison showed that fear of falling FES-I did not change in either group (BK 27.54 (7.47) to 27.36 (6.56) *p* = 0.81; and for BO 24.50 (8.24) to 24.00 (3.97) *p* = 0.06). Similarly, the ABC-scale score for fear of falling showed no significant improvement between (BK 126.18 (26.83) and BO 126.60 (35.92) *p* = 0.86) or within the groups (BK 126.72 (19.23) to 126.18 (26.83) *p* = 0.47; BO 143.66 (19.54) to 126.60 (35.92) *p* = 0.22) (Table 2).

**Table 2:** Pre- and post-intervention values for balance, gait, fear of falling, and quality of life among groups.

Variable	Boxing with kicking group (*n* = 14)				Boxing Alone group (*n* = 13)				Between groups			
	Before	*After*	*p*	*Effect-*	*Before*	*After*	*p*	*Effect-*	*Before*	*After*	*Effect-*
				*size*	*size*	*p*	*p*	*size after*
Mini-BESTest (total)*	22.60 (2.70)	25.33 (2.64)	0.02	1.02	23.09 (3.44)	25.80 (2.39)	0.01	0.91	0.75	0.53	0.18
6MWD (m) *	467.91 (76.91)	464.36 (78.07)	0.64	0.04	461.09 (73.63)	458.40 (67.87)	0.54	0.03	0.84	0.70	0.08
TUG (s)	8.03 (3.05)	9.14 (2.28)	0.06	0.41	7.74 (2.21)	8.86 (2.36)	0.007	0.48	0.94	0.72	0.12
TUG dual task (s)	8.70 (3.17)	9.65 (2.79)	0.07	0,32	8.46 (2.65)	9.33 (2.19)	0.23	0,36	0,70	0.72	0,13
FES-I (total)*	27.54 (7.47)	27.36 (6.56)	0.81	0.02	24.50 (8.24)	24.00 (3.97)	0.06	0.07	0.35	0.34	0.61
ABC-scale (total)*	126.72 (19.23)	126.18 (26.83)	0.47	0.02	143.66 (19.54)	126.60 (35.92)	0.22	0.59	0.09	0.86	0.01
PDQ-39 (total)*	22.52 (12.75)	25.93 (21.95)	0.67	0.18	26.26 (18.08)	19.01 (10.62)	0.04	0.48	0.92	0.46	0.40

*Mean (Standard deviation). Missing values differs per variable. Within group differences calculated with Wilcoxon Signed Rank. Between group differences in change scores calculated with Mann-Whitney U. Effect-size calculated with Cohen’s d. Mini-BESTest, The Mini Balance Evaluation Systems Test; 6MWD, The 6-minute walk distance; FES-I, The Falls Efficacy Scale International; ABC-scale, *The* Activities-Specific Balance Confidence Scale; PDQ-39, Parkinson’s Disease Questionnaire; TUG, Timed Up and Go Test.

### Walking ability

Overall, no significant improvement of walking ability was found between groups (BK 464.36 m (78.07) and BO 458.40 m (67.87) *p* = 0.70 or within (BK 467.91 m (76.91) to 464.36 m (78.07) *p* = 0.64; BO 461.09 m (73.63) to 458.40 m (67.87) *p* = 0.54), as measured with the 6MWD. We also found no significant between group difference for the Timed Up & Go score. However, we did see a tendency towards a decline in TUG score (longer time –worsening of function) within the BK group (8.03 s (3.05) to 9.14 s (2.28) *p* = 0.06). In the BO group (7.74s (2.21) to 8.86 s (2.36) *p* = 0.007) there was a significant decline in TUG score.

### Health status and quality of life

No significant differences related to quality of life was found between groups (*p* = 0.46) after the intervention, measured with the PDQ-39. However, the BO group showed a significant within group improvement (decrease) (26.26 (18.08) to 19.01 (10.62) *p* = 0.04) in PDQ-39.

### Conclusions

Here, we assessed the feasibility, safety, and potential effectiveness of a 10-week boxing training intervention with kicking techniques compared with an equivalent dose of boxing alone in people with PD. Our results show that both interventions were feasible and safe and showed a potential improvement in balance in people with PD. Also, both boxing with kicking techniques does not lead to additional benefits over boxing training without kicking techniques. Nevertheless, incorporating kicking techniques may be a valuable addition to the exercise therapy repertoire, by creating greater versatility and thereby assisting in long-term adherence to exercise.

The trainings were completed by 85% of the participants and most reported to be satisfied with boxing, both with and without kicking techniques. There were also no fall incidents or other adverse events during both trainings. During the trial, only four dropouts occurred for reasons unrelated to the program. Compliance and sustained adherence to prolonged exercise programs remains a critical challenge in PD [47–49]. Previous studies have shown that boxing is an engaging activity that can be sustained over the long term with additional benefits of social interaction during group trainings [18, 22, 50]. Our results add to the body of evidence showing that boxing is a feasible and pleasant exercise activity for people with PD. Using kicking techniques may be a valuable addition to the growing and versatile menu of evidence-based interventions in the field of community exercise. Personal preferences will obviously vary across individual patients and are an important motivator for exercise. Expanding the number of options may therefore assist in long-term adherence to exercise.

Although we expected that kicking techniques would further challenge the balance system, both interventions appear to be equally effective for balance outcomes. The average scores showed an increase in Mini-BESTest score of around 2-3 points for all participants, which is not clinically relevant. However, 4 people in both groups did reach a clinical relevant increase in Mini-BESTest scores of at least 4 points [38]. These results may be a reflection of the complex interplay between the many different factors that are related to balance control in PD, and we presume that balance interventions for PD will most likely need to take a multiple systems approach [25, 39–41]. Additionally, typically boxing trainings incorporate multimodal exercises including different agility, strength, and aerobic activities [17–21] that would challenge balance. Even the boxing drills, shadow boxing and speedbag drills will ultimately require dynamic weight shifting and balance tasks. Another possible factor that could have benefited balance improvements in both groups was the dual task training component of both boxing interventions. Even though, we did not see improvements in the TUG cog in both groups, the ability to perform a motor task while simultaneously engaging in a cognitively demanding task (dual-task) is critical for balance control in PD. Several studies have shown that dual task activities result in reduced balance, gait performance, and falls [39, 42, 43]. Training dual task performance has been shown to improve balance and gait outcomes [44, 45]. Finally, the training dosage of once per week for 10 weeks alongside the small sample size also potentially play a role in the limited results. Ultimately, many factors can interplay and justify why both groups improved their balance and adding kicking did not bring greater improvements.

Additionally, despite the small improvements in balance in both groups, fear of falling did not improve significantly. Improvement of balance may not directly lead to a diminished fear of falling because fear of falling is not only based on balance performance [46].

Walking ability did not show improvement in both groups. These results are not consistent with previous boxing research that showed an overall improvement on stride length and walking ability [19]. This can potentially be explained by the shorter duration and lower frequency of the intervention (10 weeks –1 time per week in this study, versus twice-a-week for 12 weeks in earlier work [18]).

We need to mention several limitations of the present study. First, the small sample size may have hampered finding statistically significant differences between groups. It was our aim to perform an exploratory pilot study aimed at evaluating the experiences/feasibility safety, and potential effects of two variants of boxing. Definitive results can therefore not be drawn. Additionally, given the type of training in group formats, the size of the groups could not be too big to assure safety.

However, the lack of a trend towards significant between group balance difference indicates that a bigger sample size would probably not have led to different results. A second limitation is that we do not have UPDRS and HY scores for our population that would better characterize the groups. We used duration since symptom onset and diagnosis as a proxy for disease severity, indicating mild to moderate disease stages in both groups. In principle, randomization should warrant an equal distribution between groups, which is a strength of the current study. We did not find any significant differences between the groups at baseline in the demographics and outcomes that we measured (Table 1), which makes us confident that both groups were comparable at baseline. Third, we did not measure the long-term effects, and consequently, no predictions can be made about any lasting effects on balance improvements for each intervention. Fourth, the frequency of training in future studies should be at least twice a week. Here, we chose a once per week frequency for feasibility reasons. Even though we believe though this dose may replicate real world access as many people with PD program different exercises each week, in the study this may limit the beneficial effects (which would be expected to be larger with a more frequent training program). Some studies that have looked at dosing effects suggest that more intense exercises come with greater benefits for people with PD [51, 52]. These limitations must be considered in future large randomized controlled trials on boxing in PD.

Ultimately, while we did not find additional benefits of kicking techniques, both boxing with and without kick techniques led to small balance improvements in people with PD. Importantly, participants were satisfied with the training, adherence was good, and no safety issues were reported. Boxing interventions have the potential to keep people with PD engaged and motivated in exercise activities over the long-term.

## Supplementary Material

Supplementary Material
