# Supplementary material for: Boxing with and without Kicking Techniques for People with Parkinson’s Disease: An Explorative Pilot Randomized Controlled Trial
Source: J Parkinsons Dis. 2022 Dec 16;12(8):2585–93. doi: 10.3233/JPD-223447 (PMC9837687; doi:10.3233/JPD-223447)
Supplement: Supplementary Material [file jpd-12-jpd223447-s001.pdf]

Supplementary Material

Boxing with and without Kicking Techniques for People with Parkinson's Disease: An Explorative Pilot Randomized Controlled Trial

Supplementary Table 1. Overview interventions

SESSION COMPONENTS	BOXING	BOXING WITH KICK-TECHNIQUES
**WARM-UP**	Same in both groups.<br>Walking through the hallway with variations in movement and speed. Alternating stepping while boxing with jab punches. Rotating trunk with hook punches and squatting on upper cuts (vertical punch).	
**MIRROR EXERCISE**	In front of the mirror, performing jab and hook punches while switching legs slowly.<br><br>Progressing weekly to faster changes in punches:<br>jabs, hooks, uppercut and the cross (opposite arm).	In front of the mirror, performing jab and hook punches while switching legs.<br><br>Progressing weekly to faster changes in punches jabs, hooks, uppercuts and the cross.<br><br>Adding kick-techniques, e.g., right foot in front of the left foot. Bringing the left knee to the front (remembering to turn hip), then placing left foot back behind the right foot. Progressing weekly with switching legs at greater speeds and kicking towards the mirror.
**PUNCH-BAGS**	Punching with jabs toward the Punchbag. Changing to the cross punch. Progressing to start with the hook and uppercut punch. Introducing punching combinations (e.g., 2 times jab, 1 hook, and 2 uppercuts). Increasing speed and the location of the punching on the Punchbag. Stacking combinations exercises.<br><br>Combining different game-like challenges to increase enjoyment (e.g., pair activities: One stand in front of the bag the other just	All the same to boxing alone group.<br><br>Including leg challenges. The first kick will be the round kick, with a bended knee, using the bag to assure balance. Next adding the kick with stretched knee. Increasing complexity with more combinations and changing speeds.<br><br>Focusing on posture by using the hook to punch and kicking the bag on different location (high to low and vice versa). Repeat 10 times for 3 sets of.

	behind him/her." Instructor" touches the "exerciser" on the arm to prompt which arm to use).<br><br>Focusing on posture by using the hook to punch the bag on different location (high to low and vice versa). Repeat 10 times for 3 series.<br><br>Adding more combinations weekly, increasing the intensity and speed of the exercises.	Adding more combinations weekly, increasing the intensity and speed of the exercises.
**ENDING (FIRE-DOWN PHASE)**	Ending sessions with free punching for 1 minute.<br><br>Cooling down with walking while moving arms in circles. Walking with big steps and whole-body movements. Moving sideways and opening both arms wide. In standing position and legs wide apart, moving arms to the side and twisting upper body from the left to the right.	Ending sessions with free punching and slow kicking for 1 minute.<br><br>Cooling down with walking while moving arms in circles. Walking with big steps and whole-body movements. Moving sideways with small kick and opening both arms wide. In standing position and legs wide apart, moving arms to the side and twisting upper body from the left to the right. Standing on 1 leg for 10 seconds, switching legs and repeating twice.
**ENDING (COOL-DOWN)**	Ending session with a relaxation exercise with soft movement, stretching and breathing.	

Questionnaire of Participants Experiences with the Training

Questionnaire

1. In welke mate heeft het boksen uw balans beïnvloedt? Mijn balans is sinds het begin van het boksen….
   O Heel veel beter (1)
   O Veel beter (2)
   O Iets (enigszins) beter (3)
   O Hetzelfde (4)
   O Iets (enigszins) slechter (5)
   O Veel slechter (6)
   O Heel veel slechter (7)

English: To what extend did boxing influence your balance? My balance is since I started boxing:

very much better (1)
much better (2)
a little better (3)
the same (4)
a little worse (5)
much worse (6)
very much worse (7)

2. Hoe tevreden bent u over het boksen (één antwoord aanvinken) ?
   O Absoluut tevreden
   O Zeer tevreden
   O Enigszins tevreden
   O Niet tevreden, niet ontevreden
   O Enigszins ontevreden
   O Zeer ontevreden
   O Absoluut ontevreden

English: How satisfied were you about the boxing training?

- *Absolutely satisfied*
- *Very satisfied*
- *A little satisfied*
- *Not satisfied, but also not unsatisfied*
- *A little unsatisfied*
- *Very unsatisfied*
- *Absolutely unsatisfied*

3. Wat motiveerde u om te gaan (en te blijven) boksen?

English: What motivated you to start (and continue) boxing?

4. Waren er ook barrières om te gaan boksen of te blijven boksen? Zo ja, welke?

English: did you experience barriers for starting or continuing boxing training? If yes, can you indicate which barriers?

5. Wat heeft het boksen u opgeleverd?

English: what were the benefits of boxing for you?

6. Zou u het andere mensen met Parkinson aanraden om te gaan boksen? Zo ja, waarom?

English: would you recommend boxing to other people with PD. If yes, why?

7. Wat is, volgens u, de meerwaarde van het gebruik van traptechnieken naast stoottechnieken?

English: What is, in your opinion, the additional value of using kicking techniques in addition to using punching techniques.
